# Is There a Link Between Green Human Resource Management and Consumer Buying Behavior? The Moderating Role of Employee Diffidence

**DOI:** 10.3389/fpsyg.2022.800936

**Published:** 2022-06-17

**Authors:** Yunxia Xiao, Rabia Younus, Wizra Saeed, Junaid Ul Haq, Xiuwen Li

**Affiliations:** ^1^Faculty of Business, City University of Macau, Taipa, Macau SAR, China; ^2^Askari Bank, Faisalabad, Pakistan; ^3^Department of Professional Psychology, Bahria University Islamabad, Islamabad, Pakistan; ^4^Faculty of Management Sciences, Riphah International University, Faisalabad, Pakistan; ^5^Business School, Hohai University, Nanjing, China

**Keywords:** green HRM, employee eco-friendly behavior, consumer behavior, diffidence, employee performance

## Abstract

Green Human Resource Management (HRM) supports promoting and incorporating sustainable development with regard to their resources. Managers and customers actively utilize the limited resources efficiently and effectively to accomplish environment-friendly goals and objectives. The study focuses on investigating the moderating role of diffidence between Green HRM, among eco-friendly behavior and Employee Performance of frontline employees of the hospitality sector. Two hundred ten individuals particapted in the research from hospitality sector with regard to examine green HRM policies of employees along with their influence on consumer buying behvaiour. Frontline employees incorporated the data on HRM performance, eco-friendly behavior, and diffidence. Besides, consumers gave their opinion on consumer buying behavior. The study’s findings revealed that Green HRM, aka Green HRM, directly impacts consumer behavior. In addition to this diffidence moderates the relationship between Green HRM and employee performance and employee eco-friendly behavior. Besides, future studies can explore the clothing and banking sector as the current study was conducted in fast food sector.

## Introduction

Studies have indicated that organizations are adopting those practices, which are more eco-friendly (Yong et al., 2019; Darvishmotevali and Altinay, 2022). This is because these practices minimize harmful impacts on the environment. Eco-friendly practices are said to be “Green practices” (Okumus et al., 2019; Yoon et al., 2020). It has become necessary for organizations to adopt eco-friendly practices that frequently involve the Human Resources (HR) function to become the potential contributor for organizational change (Mehta and Chugan, 2015). Thus, many organizations’ human resource management (HRM) function has become the driver of environmental sustainability, harmonizing green practices to reflect the eco-friendly objectives of becoming environmentally sustainable (Mandip, 2012; Rubel et al., 2021). When HRM is associated with environmental management, it refers to Green HRM (Renwick et al., 2013).

Organizations continue to convert their conventional HRM practices to Green HRM practices to enhance their environmental performance through human capital development (Álvarez Jaramillo et al., 2019; Ahmad I. et al., 2021). In addition to this, Green HRM is the transformation of HRM to become more focused upon changing employees into green employees to meet the organization’s environmental objectives (Opatha and Arulrajah, 2014) by promoting employee environmental behavior within the workplace (Renwick et al., 2013). As the organizations are shifting their HRM toward Green HRM, a change in the organization’s practices affects the employees and all of its stakeholders (Mandip, 2012). The phenomenon of employee and consumer behavior in eco-friendly practices has recently gained much attention (Tosti-Kharas et al., 2017; Xiao et al., 2021). Organizations are motivated to pursue Eco-friendly strategies, product lines, and programs (Lo et al., 2012), but the literature on green HRM is an emerging aspect (Amrutha and Geetha, 2020; Sawant, 2020).

First, recent studies have observed the impact of green HRM; at the organizational and individual levels (Shafaei et al., 2020; Ababneh, 2021) and on eco-friendly behavior and performance of employees (Lee, 2020; Gill et al., 2021; Naz et al., 2021). Literature suggested that organizational sustainability (Kalpana et al., 2020), environmental performance (Kim et al., 2019), and pro-environmental behaviors of employees (Paillé et al., 2014) have a significant impact on defining the eco-friendly behavior of employees, meanwhile, other aspects are also discussed in the literature for examining eco-friendly behavior of employees (Roscoe et al., 2019; Harasudha and Subramanian, 2020; Shafaei et al., 2020). In addition to this, most of the researches mentioned above only highlights a single aspect, either employee performance or eco-friendly behavior. Still, no analysis computed both factors collectively in a single study.

Second, the research argued that green HRM affects employees, shareholders, contractors, communities, and consumers (Mandip, 2012). Previous studies have investigated the consumer’s attitude (Han et al., 2010), behavior toward hotel’s green practices, consumer’s willingness (Manaktola and Jauhari, 2007), behavioral intention (Javed et al., 2020), consumers’ green purchase intention (Panda et al., 2020), customer environmental collaboration (Hutomo et al., 2020). Literature (Baek et al., 2006; Rajput and Gahfoor, 2020) stated that Green HRM practices has been implemented in the fast-food sector in order to enhance their customer’s experience and degree of satisfaction. Despite these researches mentioned above, efforts to explain the influence of green HRM on the antecedents of employee behavior, but no research documented the antecedents’ employee relation antecedents on consumer behavior.

Third, for the last few years, the conservation of the natural environment has been an important issue, and for this purpose, nearly every organization is switching toward environmental protection practices (Kim et al., 2019). Previous environmental management research has focused on the hotel industry in the hospitality sector (Tulsi and Ji, 2020) or the manufacturing industry (Saeed et al., 2019), or health care organizations (Mousa and Othman, 2020). Still, research has yet to focus upon the Fast food sector (Baek et al., 2006; Rajput and Gahfoor, 2020). As previously mentioned, organizations are shifting toward green HRM. Thus, this study aims to investigate the effects of Green HRM and eco-friendly (green) employees of the fast-food sector on their customer behavior.

Forth previous studies have explored that employee involvement (Chen et al., 2015) has a positive effect on performance, but reserved (Ertosun and Erdil, 2012), and shyness (Ozcelik and Barsade, 2011; Ahmad W. et al., 2021) of the employee affects their performance. Low self-esteem, introversion, and loneliness are interlinked (Kamath and Kanekar, 1993) and termed diffidence (Korn and Maggs, 2004). Diffidence is the quality of being shy and not confident in your abilities (Cambridge Dictionary, 2018). Several aspects of employee workplace loneliness are worthwhile for further study. The behavior and performance of the employee affected by the employee’s diffidence from the employee’s perspective are still unexplored.

This research aims to fill the given gaps: First, to examine Green HRM’s impact on consumer buying behavior. Second, to observe the mediating role of employee eco-friendly behavior between Green HRM and consumer buying behavior, Third, to evidence the mediating role of employee performance between Green HRM and consumer buying behavior. Fourth, to know the moderating effect of employee diffidence on the relationships between Green HRM and employee eco-friendly behavior and employee performance.

This study offers both theoretical and practical contributions. Theoretically, this research adds that Green HRM is a crucial aspect that influences the consumer buying behavior and as well as the employee behavioral outcome like employee eco-friendly behavior and employee performance. Moreover, it also contributes to the current literature that diffidence moderates the social interaction between employees and consumers. Practically, organizations should start focusing on Green HRM practices to positively impact consumer buying behavior to enhance the organization’s reputation. Moreover, organizations should focus on Green HRM to develop eco-friendly employee attitudes that can motivate and support employees in reducing their workplace diffidence.

## Literature Review

### Social Identity Theory

Social Identity theory defines as the people enjoy belonging to groups that have great reputations because it reinforces their sense of self-worth (Turner et al., 1979; Ashforth and Mael, 1989). The social identity hypothesis helps explain employer-employee relationships. Literature (Ashforth and Mael, 1989; Peterson, 2004; Karmoker et al., 2021) stated that employees who embrace their company’s beneficial activities and values leads toward becoming more committed. Besides, employees which have a favorable perception related to their working conditions leads to high involved and engage themselves in showing a higher degree of commitment level toward their growth and welfare of the organization (Stets and Burke, 2000). Besides, social identity theory portrays the employee’s higher degree of commitment and loyalty toward their organizations (Ashforth and Mael, 2004).

### Social Exchange Theory

Social exchange theory elaborated the conduct between two or more parties in terms of conducting social interaction for the sole objective of exchanging goods. Besides, further illustrates that actions are contingent in terms of rewarding others in accordance with their behavior (Blau, 1968). For that reason, Social Exchange theory tends to emphasize the dependence among people in a relationship (Lee and Cadogan, 2009). Hence, organizations lead to promising their employees with regard to accomplishing their assigned tasks efficiently and effectively as it will result in fruitful outcomes (Yin, 2018).

### Green Human Resource Management and Consumer Buying Behavior

Green HRM is defined as the process of making green employees with the use of green HR policy and practices, and this is for the benefit of individuals, community, business, society, and the whole planet (Opatha and Arulrajah, 2014). Environmental sustainability is critical for organizations, employees, governments, the general public, and marketers (DoPaço et al., 2019). Research studies have examined which indicates that the organizations are adopting the environmental protection practices which have given rise to green HRM (Sarkar, 2008). This movement has also given rise to the expansion of a new segment, i-e, environmentally concerned consumers, also known as green consumers, engaged in green behaviors (Finisterra do Paço and Raposo, 2010). Consumer buying behavior is defined as “the buying behavior of final consumers individuals and households that buy goods and services for personal consumption” (Kotler and Armstrong, 2010, p.159; Rao et al., 2021). Researchers (Cherian and Jacob, 2012; Woo and Kim, 2019; Rodrigues et al., 2021) identified factors influencing consumers’ buying behavior toward environmentally friendly services. These factors include the type of green communication from the organization, environmental practices made by the organization, the validity, and availability of information, and the variety of green services served by the providers or producers. The two factors i-e green communication from the organization, environmental practices, and a variety of green services served by the providers or producers, are those factors that are presented directly by the green employees to the consumers. Besides, the AMO theory (Appelbaum et al., 2000; Renwick et al., 2013) proposes the impact of GHRM on the performance of their employees in terms of their abilities, motivation, and opportunity of using incorporate green objectives while serving their customers accordingly. So it can be assumed that consumers’ buying behavior can be influenced by these two factors mentioned above submitted by green employees, and as we know, employees are made green through green HRM (Opatha and Arulrajah, 2014). Hence, it is said that green HRM can be a factor that can influence consumers’ buying behavior. Based on the discussion mentioned above, this study proposed the following hypothesis:

**H1:** *Green HRM has a positive impact on Consumer Buying Behavior.*

### Green Human Resource Management and Employee Eco-Friendly Behavior

One of the main purposes of green HRM is to develop environmental responsibility and sensitivity in employees to become aware of how their behaviors (in-role and extra-role) affect the environment (Gilal et al., 2019). The research on employee environmental behavior can be considered part of organizational citizenship behavior (Lülfs and Hahn, 2013). Besides, it refers to the voluntarily acted pro-social behaviors of individuals in the work environment that can also go beyond that when employees represent their organization to consumers (Organ et al., 2005). A cooperative pattern of conduct is reflected that is supererogatory and represents employee voluntary willingness to make extra efforts and do their part in the welfare and benefit of the organization and its stakeholders (Raineri and Paillé, 2016). For understanding the eco-friendly behavior in the workplace, the organizational citizenship behavior for the environment (OCBE) has acquired much attention (Paillé et al., 2013). Paillé et al. (2014) proposed that Green HRM has a positive and significant relationship with employee’s OCBE. Kim et al. (2019) proposed that green HRM has a positive impact on employee eco-friendly behavior, and it includes some actions such as water usage, waste usage, and energy usage. According to the discussion mentioned above, this study proposed the following hypothesis:

**H2:** *Green HRM has a positive impact on Employee’s Eco-friendly behavior.*

### Green Human Resource Management and Employee Performance

Employee job performance is an important part of every organization (Elnaga and Imran, 2013). An organization should develop green rewards, green abilities, motivate employees to increase their performance (Renwick et al., 2013). Employee job performance is defined as the employees’ use of knowledge and skill in bringing about products or services that contribute to the technical foundation of the organization (Evangeline and Thavakumar, 2015). Employee’s environmental performance includes continuous improvement, stakeholder perception, independent audits, recycling performance, waste reduction, cost-saving, and resource consumption. HR managers achieve these performance objectives by recruiting, appraising, training, and incentivizing the cognizant environmental workforce (Harvey et al., 2013). Based on environmental goals, employee performance evaluation is a key role of HR managers (Roscoe et al., 2019). Paillé et al. (2013) proposed that green HRM has a positive impact on employee performance. By adopting green HRM, organizations have green evaluation systems. The HR managers can discuss with their working employees about achieving environmental objectives, ideas for waste minimization and performance enhancements, and improvements. In light of the above discussion, this study proposes the following hypothesis:

**H3:** *Green HRM has a positive impact on Employee Performance.*

### Employee Eco-Friendly Behavior and Consumer Buying Behavior

As employees are shifting toward green practices, consumers are also becoming keenly aware of green issues such as a threat to natural resources, global warming, and pollution; thus, they consider these issues when making green purchasing (Young et al., 2010). The employees are the key agents of the organization that present its main products and services to consumers. Consumers can’t see the whole organization’s procedures, and they only have contact with their employees and consumers make their perceptions about the whole organization from the behavior of the employees. Based on a literature review, an employee’s eco-friendly behavior is referred to as the extra-role behavior that is not formally required from an employee’s job and is not appraised by the reward system (Chou, 2014). Still, these extra-role behaviors are noticed by consumers (Zhao et al., 2018). Previous research has also shown that employee extra-role behavior displayed during the delivery of services is valued and perceived by customers (Bell and Menguc, 2002) and a high level of employee extra-role behavior tends to lead to a high service quality impression among customers (Bell and Menguc, 2002) which can affect their buying behavior. Yoon and Suh (2003) proposed that the organizations must encourage these employees’ discretional voluntary behaviors because they can enhance the contact between employees and consumers. This contact can help shape consumers’ intention and behavior to remain consumers of the same organization (Castro et al., 2004). Employees’ eco-friendly behavior tends to attract a customer in terms of their buying behavior as the management seeks to fulfill their desire accordingly (Xess et al., 2021). Besides, literature (Laroche et al., 2001; Han et al., 2009) stated that customer’s behavior gets influenced by the eco-friendly practices, especially in the hospitality sector Based on these assumptions, the authors of this study postulated that:

**H4:** *Employee’s Eco-friendly Behavior has a positive impact on Consumer Buying Behavior.*

### Employee Performance and Consumer Buying Behavior

Consumers are the most salient groups where frontline employees interact (Korschun et al., 2014; Butt et al., 2017). Consumers evaluate firms according to the consumer-employee dyadic relationship (Zhou et al., 2014). Consumers experience the whole service offered by the organization not only from the physical environment but also through their employees, and these interactions shape the buying behavior of the consumers. Companies are taking measures to enhance employee performance (Jain and Sharma, 2019). If the employee’s performance is good, it will make the consumers satisfied, which will improve consumers’ buying behavior, and increase the level of satisfaction.

Positive interaction between employees and customers increases customer loyalty (Berry and Parasuraman, 1991). Liao and Chuang (2004) proposed that employee service performance positively affects customer outcomes, while (Netemeyer et al., 2005) proposed that employee performance plays a key role in influencing consumers to purchase again from an organization. Literature (Yoon et al., 2016; Du Preez et al., 2017) suggested that employees tend to perform eco-friendly practices while interacting with customers which leads to establishing trust which in turn influences the behavior to purchase. Moreover, green practices encourage employees to ethically understands and fulfill their consumer’s needs and desires in order to retain them for a longer period. So it can be said that employee performance is not only noticed and appraised by the organization but also has a positive effect on consumers’ buying behavior. This study proposed the following hypothesis in the light of the above discussion:

**H5:** *Employee Job Performance has a positive impact on Consumer Buying Behavior.*

### Mediating Role of Employee Eco-Friendly Behavior

Organizations have an HRM department for hiring and selection, training and development, reward, compensation, and performance improvements of their employees (Renwick et al., 2013). The practices of Green HRM can improve employees’ ecological behavior to deliberately enhance the firm’s performance (Pham et al., 2019), which can enhance the consumers’ willingness to pay more for a firm’s green measures (Kang et al., 2012). Green HRM is making employees eco-friendly attitudes and behavior of the consumers are influenced by the attitude and behavior of the employees. The organizations implement green practices through recruitment and training of green employees by adopting Green HRM, which ultimately positively affects consumers’ buying behaviors due to these practices. This study proposed the following hypothesis:

**H6:** *Employee Eco-friendly Behavior will mediate the relationship between Green HRM and Consumer Buying Behavior.*

### Mediating Role of Employee Performance

The shift of responsibility toward the society and environment has been the concern of organizations, scholars, and government (Rajnoha and Lesníková, 2016). It gave rise to consumers’ green purchasing behaviors (Liobikienė et al., 2016; Sharma et al., 2021). The company’s senior management is applying environmentally responsible management due to the increasing pressure on environmental protection (Porter and Van der Linde, 1995). Companies are adopting the environmental management practice of Green HRM to implement green practices and develop employee awareness which will increase the economic performance and employee performance through energy consumption and water savings, which will increase consumer satisfaction (Molina-Azorín et al., 2015). The organization’s Green HRM enhances the performance of the employees related to environmental protection (Chen et al., 2015). The performance of the employee is shown among other employees and in front of the consumers. The employee’s performance is appraised and rewarded by the HRM, and his performance will influence the consumer’s buying behavior. In the light of the discussion above, this study proposed the following hypothesis:

**H7:** *Employee Performance will mediate the relationship between Green HRM and Consumer Buying behavior.*

### Moderating Role of Diffidence

Interpersonal communication is essential for an organization because a better relationship can be developed between employees and consumers, better expressing and receiving consumer needs (Burachati, 2010). It has been observed in previous research that employees in an organization are undergoing inner conflicts, negative mindset conservatism, lack confidence (Asadullah et al., 2019; Bukhari et al., 2021), possess loneliness (Çetin, 2018), and exhibit shyness (Zhao et al., 2013). All the factors mentioned above are psychologically disturbing to the individual and termed as Diffidence. Employees must stimulate an environment where people can speak up and develop better communication (Asadullah et al., 2019). Still, diffidence interferes with good relations with others (Yadav and Chadha, 2017). Diffidence is referred to as the hindrance, discomfort, and feeling of shy and reserved in social situations. Due to diffidence, employees will not be able to perform their duties properly for which they are trained, which could affect their interaction with consumers and ultimately affect the entire organization. If employee diffidence increases, it can moderate Green HRM’s relationship with eco-friendly employee behavior and performance. Hence, this study proposes that:

**H8 (a):** *Diffidence moderates the relationship bet*ween *Green HRM and Employee Ecofriendly Behavior.*

**H8 (b):** *Diffidence moderates the relationship between Green HRM and Employee Performance.*

### Research Design and Context

Data for this study was obtained from the frontline employees and customers of different fast-food chains. Frontline employees and customers visiting fast-food chains were selected for this study because globally, the hospitality sector is considered agents working to promote sustainability (Huang and Lee, 2014). This research is like a blade of a sword that cuts from both sides. Similarly, this research documented both employee and consumer end from the frontline employees of fast-food chains and consumer who visits these fast-food restaurants. Shebl et al. (2021) stated that fast food restaurants are visited by customers on a larger scale, for that purpose, KFC, McDonalds, PizzaHut and Hardees were used while collecting data for the research.

During service encounter, the consumer interacts with first-line employees (and observe his performance) which ultimately affects consumer behavior. Instance, Zhao et al. (2018) and Kim et al. (2019) followed the similar design for conducting their research.

### Data Collection

Data was collected from 210 respondents by adopting a questionnaire technique. A questionnaire has two parts: the first was for the frontline employees, and the second was for the customers. Both parts of the questionnaire comprised three sections. The first section briefly described the purpose of the data collection and assurance of confidentiality of all responses. The second section included information on the respondents’ demographics to verify the study respondents’ diversity. The third section consisted of predefined, closed-ended questions about the research. Researcher applied convenience data collection technique due to the time constraints and pandemic restrictions.

### Sample Size

Data was collected from the hospitality sector especially from restaurants. The main focus of the study was the fast-food sector within the hospitality sector. Customers and frontline employees were contacted and informed about the study’s objectives. Favorable support was obtained to administer, the questionnaires and all management confirmed that their organizations had established programs involving employees and their green behavior. A contact person from every fast-food chain was identified to provide customers who agreed to assist with data collection from each brand. The questionnaire was distributed and collected directly from the frontline employees and customers during their visit to that particular brand. Complete anonymity and secrecy of the respondents were assured as the management and customers had no access to the completed responses. The researchers ensured that the frontline employees and customers filled their respective parts of the questionnaire separately at separate timings to maintain secrecy. After the data collection, the researchers paired each questionnaire of frontline employees with their customers by asking for the customer’s feedback about their facilitated frontline employee in both parts of the questionnaire. Sixty questionnaires were distributed at each fast-food brand, the total number of 300 questionnaires was distributed. Data of 210 respondents was the appropriate sample size because Hair et al. (1998) stated that the sample size could be 5 times the number of items, but in this study 210 sample size was considered for better results, and the opinion of more respondents. Moreover, the data were collected from those respondents who have used the services of fast food in order to enhance the validity and reliability of the research. Besides, experienced respondents can get a better understanding of the purpose along with the situation for giving their opinion accordingly. Meanwhile, Convenient Sampling techniques were used for the data collection process as the approach emphasizes stated precise outcomes for the proposed variables in the framework. Additionally, the data collected with the help of convenience approach do not exceed beyond the variables.

Out of 360 frontline employees, only 255 participated in this survey from all six fast-food brands because of their visits and personal availability. Removing 45 incomplete questionnaires, 210 were useable for the study representing a response rate of 58.33%. The detail of these respondents can be seen in Tables 1, 2.

**TABLE 1:** Demographics of front line managers (employees).

Demographics variables	Categories	Frequency (*n*)	Percentage
Age	31–35	21	10.0
	36–40	114	54.3
	41-above	75	35.7
Gender	Male	130	61.9
	Female	80	38.1
Education	Intermediate	4	1.9
	Bachelors	153	72.9
	MPhil	53	25.2
Restaurants you are working in	Fast food	113	53.8
	Cultural food	97	46.2

**TABLE 2:** Demographics of visitors (consumers).

Demographics variables	Categories	Frequency (*n*)	Percentage
Age	16–20	73	34.8
	21–25	82	39.0
	26–30	54	25.7
	31-above	1	0.5
Gender	Male	111	52.9
	Female	99	47.1
Education	Inter	104	49.5
	Bachelors	103	49.0
	MS/M.Phil	3	1.4
Restaurant you often visit	Fast food	113	53.8
	Cultural	97	46.2

*Respondents were comprised of two sets. First set was the visitors and the second set was front line managers. Researcher targeted the first set and found the people of having age 16–31. Both genders participated in this research. Researcher focused on the educated respondents to collect a fine set of data. Similarly, in second set of data researcher targeted the managers of restaurants, the ages of respondent were lied in the age spam of 31–40. While the education of the researchers were intermediate to MPhil level.*

### Measurements

Green HRM, employee eco-friendly behavior, diffidence, and employee performance was rated by the frontline members of different fast food brands Green HRM and Eco-friendly behavior were measured with six and seven items scale, respectively (Kim et al., 2019). Diffidence was measured using the thirteen-item scale of shyness (Hopko et al., 2005). Each item of the Green HRM, employee eco-friendly behavior, and diffidence was measured using the 5-points Likert scale ranging from strongly disagree to strongly agree. Employee performance was measured using the six-item scale adapted from Salanova et al. (2005). Each item was measured using the five-point rating scale ranging from completely disagree to agree.

The consumers of different fast-foods’ industry rated consumer buying behavior and it was measured using the two-item scale adapted from Chan and Lau (2000), and each item was measured using five-point scales ranging from never to always.

## Results and Findings

### Data Analyses

Data set of 210 responses were entered in Statistical Package for Social Sciences (SPSS). Initial screening was done through normality, reliability, and validity. Normality was observed with Skewness and Kurtosis. Findings approved that all the items are within the normal range of + 3 (Hair et al., 1995). Reliability was measured through Cronbach’s alpha, internal consistency among the variable items, and composite reliability in the confirmatory analysis. The values of all the reliability tests were in acceptable ranges and can be viewed in Table 3. Confirmatory Factor Analysis (CFA) was carried using AMOS. Validity was measured through convergent and discriminant validity. Initially, five-factor model measurement fit indexes were observed and found within acceptable ranges. Model fit measures were observed through chi-square, degree of freedom, and CMIN/DF, and values were 329.503, 287, and 1.148, respectively. Absolute fit indexes were measured through RMSEA = 0.027, GFI = 0.895 and AGFI = 0.872. Incremental fit measures were estimated through CFI = 0.983, NFI = 0.885 and IFI = 0.983. Researchers proposed factor loading and average variance extracted for good convergent validity measures. The values of factor loading and average variance extracted should be greater than 0.5 (Fornell and Larcker, 1981). All the values were above the threshold level, as shown in Table 3.

**TABLE 3:** Reliability and validity.

Variables	Items No.	FL	CR	Cronbach alpha	AVE
Green HRM	GHRM 1	Deleted	0.83	0.83	0.55
	GHRM 2	Deleted			
	GHRM 3	0.70			
	GHRM 4	0.74			
	GHRM 5	0.81			
	GHRM 6	0.72			
Employee eco-friendly behavior	EEB 1	0.71	0.88	0.88	0.51
	EEB 2	0.72			
	EEB 3	0.66			
	EEB 4	0.73			
	EEB 5	0.60			
	EEB 6	0.78			
	EEB 7	0.75			
Diffidence	D 1	0.47	0.90	0.90	0.52
	D 2	0.81			
	D 3	Deleted			
	D 4	0.51			
	D 5	0.75			
	D 6	Deleted			
	D 7	0.78			
	D 8	0.74			
	D 9	Deleted			
	D 10	0.72			
	D 11	0.77			
	D 12	Deleted			
	D 13	0.85			
Employee performance	EP 1	0.74	0.85	0.85	0.59
	EP 2	Deleted			
	EP 3	Deleted			
	EP 4	0.80			
	EP 5	0.72			
	EP 6	0.81			
Consumer buying behavior	CBB 1	0.79	0.77	0.77	0.63
	CBB 2	0.79			

*FL, factor Loading; AVE, average variance extracted; CR, composite reliability; GHRM, Green HRM; EEB, employee eco-friendly behaviour; D, diffidence; EP, employee performance; CBB, consumer buying behavior.*

Researcher deleted some of the items to develop a good model fit and for the reduction of the cross loading. The development of the good model fit indices is the requirement to develop a good reliability and validity analysis (Hair et al., 2014). So this is why researcher deleted the some of the items from different scales.

Discriminant validity was examined through the correlation between the variables of the study, and the square root of AVE should be greater than its peer values (Fornell and Larcker, 1981; Ul Haq and Bonn, 2018). All the values were in the acceptable range and it can be observed in Table 4.

**TABLE 4:** Discriminant validity.

	CR	AVE	EP	GHRM	CBB	EEB	D
EP	0.854	0.594	**0.771**				
GHRM	0.830	0.551	0.364	**0.742**			
CBB	0.772	0.628	0.455	0.560	**0.793**		
EEB	0.877	0.506	0.445	0.598	0.598	**0.711**	
D	0.904	0.518	0.106	0.119	0.041	0.179	**0.720**

*GHRM, Green HRM; EEB, employee eco-friendly behavior; D, diffidence; EP, employee performance; CCB, consumer buying behavior. All diagonal bold values are the square root of AVE.*

### Biases and Multi-Collinearity

Biases were observed through common method variance using Herman’s single factor test as proposed by Podsakoff et al. (2003). This estimation was done because of the cross-sectional data set. The study’s authors observed the common method variance value as 24.78%, demonstrating that no biasness existed in the data set. Multi-collinearity was observed through the variance inflation factor test in SPSS. All factors had variance ranges from 1.02 to 1.60, which is under the acceptable range of 3. Hence, it can be predicted that there is no multicollinearity issue in the data set for the proposed conceptual framework.

### Hypotheses Testing

**FIGURE 1:**
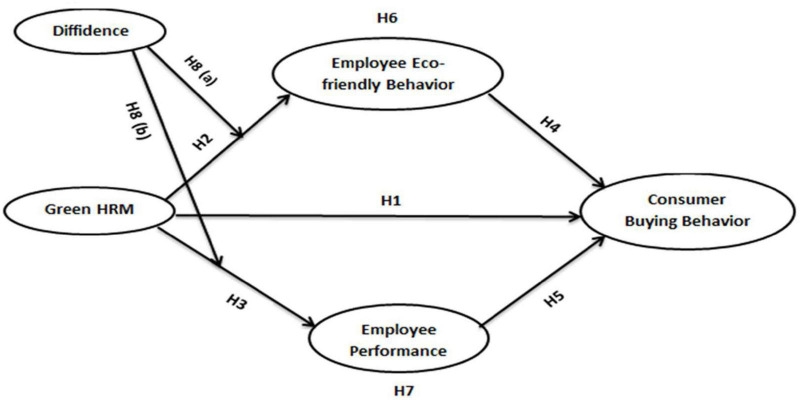
Conceptual framework.

Hypotheses testing was estimated using the partial-structural equation modeling technique in AMOS. Initially, the structural model fit indexes were observed. All the values of model fit were found in acceptable range that is CMIN/DF = 16.018, GFI = 0.964, AGFI = 0.644, CFI = 0.915, NFI = 0.912, TLI = 0.490 and RMSEA = 0.268. Furthermore, the approved conceptual model can be viewed in Figure 1. Standardized estimated values of hypotheses can be seen in Table 5 along with mediation results.

**TABLE 5:** Hypotheses testing.

Hypotheses	Description	Standardized estimate	Supported/not supported
H1	GHRM → CBB	0.24*	Supported
H2	GHRM → EEB	0.52*	Supported
H3	GHRM → EP	0.31*	Supported
H4	EEB → CBB	0.30*	Supported
H5	EP → CBB	0.19**	Supported
H6	GHRM → EEB → CBB	0.15*	Partial Mediation
H7	GHRM → EP → CBB	0.06**	Partial Mediation

*Significance level < 0.001, **Significance level < 0.01. GHRM, Green HRM; EEB, employee eco-friendly behavior; D, diffidence; EP, employee performance; CCB, consumer buying behavior.

Researcher testified the mediation analysis by applying the amos plugin (My specific indirect effects estimands) this plugin eases the researcher to conclude mediation analysis directly without using bootstrapings.

### Moderation Testing

**FIGURE 2:**
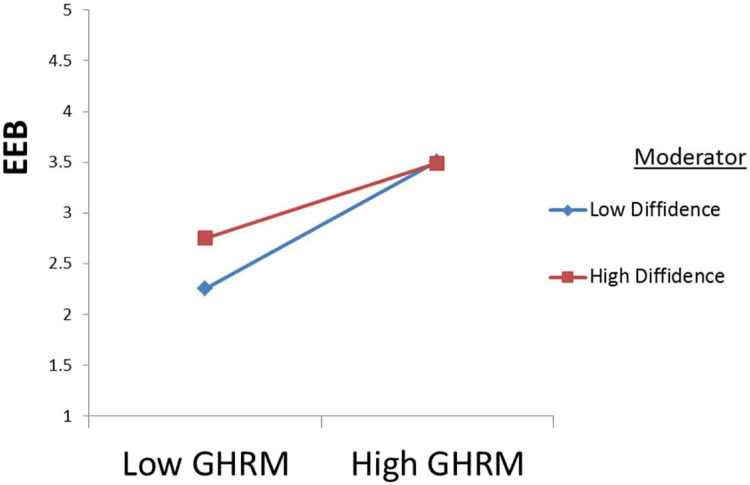
H8(a).

**FIGURE 3:**
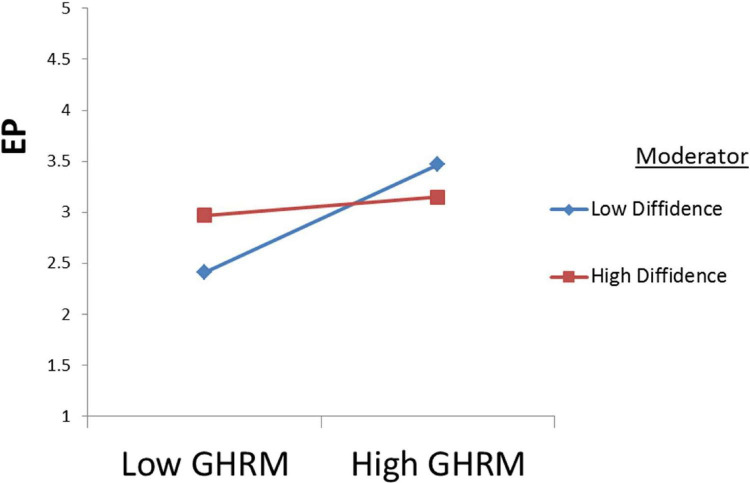
H8(b).

The moderation was checked through Partial-SEM in AMOS by following the path of researcher (Bukhari et al., 2021). It was proposed that employee diffidence moderates the relation between Green HRM and employee eco-friendly behavior and Green HRM and employee performance. Moderation was checked by finding the Z-score of these variables and the interaction term in SPSS. To find the Z score, the variables were first computed then the Z score was determined. Secondly, the interaction term was determined by multiplying the Z score of independent and moderator. Lastly, Partial-SEM was run in AMOS. The results revealed that Diffidence dampened the relationship between Green HRM and employee eco-friendly behavior and Green HRM and employee performance supporting H8(a) and H8(b) as shown in Figures 2, 3.

## Discussion and Implications

Based on those study responses of the respondents, all hypotheses were accepted. The conceptual framework of this study contributed theoretically and empirically for academicians and was found to be practical for management.

### Implications for Academicians

This research added to the scant amount of existing literature for academicians. The findings of this study offer a significant contribution to the literature. Firstly, it contributes to the green HRM literature by adding supportable evidence of the relationship between organizations’ green HRM and consumer buying behavior. Secondly, this study also contributed to the literature of the diffidence that employee’s diffidence influences social interaction between employee and consumer, affecting the employee’s behavior and performance. Employee shyness, loneliness, lack of confidence, etc., have been studied in the past, but this is the first study that has focused on the combination of these factors i-e diffidence. Thirdly, it also contributes to the literature to the indirect effect of Green HRM on consumer buying behavior that has never been investigated in terms of mediators such as employee eco-friendly performance and behavior. The study provides an important understanding of the relationship between Green HRM and employee green behavior and performance by hypothesizing the moderating effect of employee diffidence.

### Implications for Practitioners

Results suggest that managerial practitioners can achieve their sustainable goals more efficiently and effectively by following this study’s findings. *Green HRM* is the first step organizations should embrace involving environmental sustainability. The firms should focus on their green HRM practices and provide green training, initiatives, and green rewards to employees to get them engaged in green practices. Moreover, such practices can influence consumers’ buying behavior and, in return, can influence and increase the organization’s environmental environment social image. *Employee Eco-friendly* is the extra-role behavior of employees. If the organizations want to encourage eco-friendly behavior at the individual level, they can do so by implementing HRM activities, practices, and policies directed toward the environment. *Employee performance* is measured by HRM performance evaluation. Organizations can improve their performance management system by aligning their performance management system with environmental management objectives which can also act as performance indicators during evaluation by setting such eco-objectives for the employees to achieve those and improve their performance. *Diffidence* or shyness of employees can be reduced by motivating them for eco-friendly behavior. The lower the diffidence, the higher employees will exhibit eco-friendly behavior. Organizations should help, inspire, and give support to the employees and give training to them on how they can minimize their diffidence in social interactions and gatherings in the workplace.

## Conclusion, Limitations, and Future Research

This study presented an approved hierarchical model for consumer behavior. This model observed that sustainable consumer behavior can be manifested through the employment of Green HRM, eco-friendly employee behavior, and employee performance. Green HRM positively and significantly contributed to employee eco-friendly behavior and employee performance. Ecofriendly behavior and performance of employees simultaneously support consumer behavior. In addition to this, it has also been observed that Green HRM makes a direct and indirect effect (through employee eco-friendly behavior and employee performance) upon consumer behavior. Diffidence has been proven to be a barrier for employees to perform their green motives, and especially involving their eco-friendly behavior and performance.

This study has several limitations that will help guide future research in this area. First, this study considered information from respondents in a hospitality industry in a developing country. Although the cities are diverse in terms of demographics, behavior toward sustainable models can vary. Future studies are encouraged to apply the same model using different countries in different demographics so that intra-city and intra-country comparison can be estimated to determine the model’s acceptability. Second, this study collected the responses of employees and consumers of fast-food and observed their behavior for green policies of fast-food sector. Future researchers can observe the same model for educational institutions, banks, and other services sectors. Third, this study addressed diffidence as only one barrier regarding employees and Green HRM. Future studies should consider other barriers such as communication skills, short-term focus, resistance to change, etc.

## Data Availability Statement

The raw data supporting the conclusions of this article will be made available by the authors, without undue reservation.

## Ethics Statement

The studies involving human participants were reviewed and approved by this study involving human participants was reviewed and approved by the Ethics Committee of the Department of Management Sciences, Riphah International University Islamabad, Faisalabad Campus, Faisalabad, Pakistan. The participants provided their written informed consent to participate in this study. The patients/participants provided their written informed consent to participate in this study.

## Author Contributions

All authors listed have made a substantial, direct, and intellectual contribution to the work, and approved it for publication.

## Conflict of Interest

The authors declare that the research was conducted in the absence of any commercial or financial relationships that could be construed as a potential conflict of interest.

## Publisher’s Note

All claims expressed in this article are solely those of the authors and do not necessarily represent those of their affiliated organizations, or those of the publisher, the editors and the reviewers. Any product that may be evaluated in this article, or claim that may be made by its manufacturer, is not guaranteed or endorsed by the publisher.
